# 415. Misdiagnosis of multisystem inflammatory syndrome in children: A diagnostic challenge

**DOI:** 10.1093/ofid/ofad500.485

**Published:** 2023-11-27

**Authors:** Gülhadiye Avcu, Aslı Arslan, Sema Yildirim Arslan, Zumrut Sahbudak, Caner Turan, Irem Ersayoglu, Kübra Cebeci, Zafer Kurugol, Ferda Ozkınay

**Affiliations:** ege university, izmir, Izmir, Turkey; Ege University School of Medicine Department of Pediatric Infectious Disease, İzmir, Izmir, Turkey; Division of Infectious Disease, Department of Pediatrics, İzmir, Izmir, Turkey; Ege Univesity, izmir, Izmir, Turkey; Ege Univesity, izmir, Izmir, Turkey; Ege Univesity, izmir, Izmir, Turkey; Ege Univesity, izmir, Izmir, Turkey; Ege Univesity, izmir, Izmir, Turkey; Ege Univesity, izmir, Izmir, Turkey

## Abstract

**Background:**

As the COVID-19 pandemic continues, multisystem inflammatory syndrome in children (MIS-C) maintains its importance in the differential diagnosis of common febrile diseases. MIS-C should be promptly diagnosed because corticosteroid and/or intravenous immunoglobulin treatment can prevent severe clinical outcomes. In this study, we aimed to evaluate clinical presentation, diagnostic parameters and management of MIS-C and compare its clinical features of common febrile disease.

**Methods:**

This study was conducted at a tertiary-level hospital between December 2020 and October 2022. One hundred and six children who were initially considered to have MIS-C disease were included in the study. During the follow-up period in the hospital, when the clinical and laboratory findings were re-evaluated, 38 out of 106 children were diagnosed differently. The clinical and laboratory findings of 68 children followed up with the diagnosis of MIS-C and 38 children who were initially misdiagnosed as MIS-C but with different final diagnoses were retrospectively compared.

**Results:**

We identified 68 patients with MIS-C and 38 patients misdiagnosed as MIS-C during the study period. Infectious causes (71%), predominantly bacterial origin, were the most frequently confused conditions with MIS-C. Patients with MIS-C were older and had a more severe clinical course with high rates of respiratory distress, shock, and paediatric intensive care unit admission. While rash and conjunctivitis were more common among patients with MIS-C, cough, abdominal pain, diarrhoea were observed more frequently in patients misdiagnosed as MIS-C. Lower absolute lymphocyte counts, platelet counts and higher C-reactive protein and fibrinogen levels, pathological findings on echocardiography were the distinctive laboratory parameters for MIS-C. Multivariate analysis showed that older age, presence of conjunctivitis, high level of serum CRP and lower platelets were the most discriminative predictors for the diagnosis of MIS-C.

**Conclusion:**

There are still no specific findings to diagnose MIS-C, which therefore can be confused with different clinical conditions. Further data are needed to assist the clinician in the differential diagnosis of MIS-C and the diagnostic criteria should be updated over time.

**Disclosures:**

**All Authors**: No reported disclosures

